# Invasive fungal infections among critically ill children: Epidemiology, risk factors and outcomes

**DOI:** 10.7196/AJTCCM.2018.v24i1.172

**Published:** 2018-04-03

**Authors:** S T Hlophe, N P Govender, R Masekela

**Affiliations:** 1 Department of Paediatrics and Child Health, Nelson R Mandela School of Clinical Medicine, University of KwaZulu-Natal, Durban, South Africa; 2 Centre for Healthcare-associated Infections, Antimicrobial Resistance and Mycoses, National Institute for Communicable Diseases, National Health Laboratory Service; and School of Pathology, Faculty of Health Sciences, University of the Witwatersrand, Johannesburg, South Africa

**Keywords:** invasive fungal infections, PICU, risk factors

## Abstract

Critically ill children are at high risk of developing invasive fungal infection in a paediatric intensive care unit. This is due to the vulnerability of these children and invasive nature of the care provided.

## Background

Paediatric intensive care units (PICUs) are often multidisciplinary, admitting both medical and surgical patients. The innate vulnerability of critically ill children and the invasive nature of care provided in a PICU make this a high-risk setting for healthcare-associated infections (HAIs). HAIs are an important cause of morbidity and mortality in this population, yet are potentially preventable. Dramowski *et al*.^[1]^ reported an HAI prevalence of 16.5% at Tygerberg Children’s Hospital, Cape Town, during a 6-month period in 2015. Similarly, Spicer *et al*.^[2]^ reported a prevalence of 20.4% and an incidence of 15.3 cases per 100 PICU admissions over a 2-year period at Grey’s Hospital, Pietermaritzburg. The overall mortality attributed to paediatric HAIs has been estimated at 11%.^[3]^ Invasive fungal infections are among the top four causes of paediatric HAIs.^[4]^ This review is aimed at describing the epidemiology, risk factors and mortality associated with invasive fungal infections among critically ill children admitted to PICUs.

## Organisms

Fungi are ubiquitous organisms that live as environmental saprophytes or as commensal microorganisms of humans and animals.^[5,6]^ Medically important fungi are commonly opportunists and rarely primary pathogens in exposed immune-competent subjects. However, when encountered in the context of a PICU they can cause opportunistic infections. Species in the genetically diverse genus *Candida* commonly cause invasive disease among critically ill children; *Aspergillus* spp., mucocutaneous moulds and other rarer emerging fungi occasionally cause disease.^[5,6]^ Invasive infections caused by *Candida* spp. and *Aspergillus* spp. are associated with high mortality and morbidity as well as high healthcare costs. In this review, we therefore focus only on invasive disease caused by these two pathogens.

## Candidaemia and invasive candidiasis

*Candida* spp. are the leading cause of invasive fungal infections in hospitalised children and are the third most common isolates recovered from paediatric cases of healthcare-associated bloodstream infection in the United States.^[7,4]^ Spicer *et al*.^[2]^ found that 23.8% of all the HAIs at Grey’s Hospital were bloodstream infections. Dramowski *et al*.^[1]^ reported that 6% of HAIs were caused by *Candida* spp. In children, Candidaemia is associated with prolonged hospital stay (median 21 days) and increased costs.^[4]^ *Candida* *albicans* is the most common invasive species in the paediatric population, causing 55% of cases.^[4]^ *Candida* *parapsilosis* and *Candida* *tropicalis* are other common species, which contribute to 17.5% and 10% of the burden of invasive disease, respectively.^[4]^ *Candida* *glabrata* and *Candida* *krusei* are less frequently cultured but may be encountered in specialist units.^[8]^ Distinguishing *Candida* colonisation from infection is not always straightforward. Invasive fungal infection is defined as a positive culture from either blood or sterile sites, together with clinical or laboratory evidence of a systemic inflammatory host response. In contrast, colonisation typically occurs in the absence of such a response in cultures from non-sterile sites such as the respiratory tract.^[9,10]^ *Candida* isolation from respiratory secretions alone should never prompt treatment.^[11]^ Invasive candidiasis is distinguished from Candidaemia by clinical, radiological, microbiological or histological evidence of disseminated foci of infection, e.g. splenic/hepatic abscesses or endophthalmitis.^[11]^

Invasive *Candida* infection has a reported attributable mortality in children of between 20% and 30%.^[4]^ In most studies, *Candida* is one of the predominant causative agents for sepsis in hospitalised children, together with coagulase-negative staphylococci, enterococci and *Staphylococcus aureus*. ^[4]^ Admission to the PICU is a risk factor for invasive *Candida* infection.^[5]^ In another Tygerberg Hospital study, the pathogens associated with the highest mortality from bloodstream infections were *Acinetobacter* spp. (38%; *n*=30/78), followed by *Candida* spp. (31%; *n*=20/65) and *Escherichia coli* (24%; *n*=23/97).^[12]^ In the same study, all 21 *C. albicans* isolates were susceptible to fluconazole, whereas 22 of the 44 isolates other than *C. albicans* were resistant to fluconazole.^[12]^

Among all risk groups, *Candida* colonisation is an independent risk factor for infection and precedes invasive infection in most cases.^[9]^ The risk of infection increases with the number of colonised sites and is dependent on the colonising species. *Candida* colonisation in the gastrointestinal tract has been reported as a significant risk factor for invasive candidiasis in many studies.^[13]^ Other reported risk factors include:^[4,12]^

- the presence of a central venous catheter
- total parenteral nutrition
- a pre-existing bacterial infection
- immunocompromised status of the host
- recent surgery
- dialysis
- prolonged use of vancomycin
- administration of antimicrobial agents against Gram-negative bacteria
- mechanical ventilation

Various scoring systems have been developed to aid the differentiation between infection and colonisation and these systems aid in identifying patients at risk of developing infection. The system used for the *Candida* score and index, developed for critically ill adults, allocates points for each of the following criteria:^[4,14,15]^

- total parenteral nutrition = 1 point
- surgery on ICU admission = 1 point
- multifocal *Candida* colonisation = 1 point
- severe sepsis = 2 points

The *Candida* score is a useful tool to differentiate critically ill patients, who would benefit from early antifungal treatment (score >3), from those in whom invasive candidiasis is highly improbable (score ≤3).^[15]^ In a large cohort of non-neutropenic, critically ill patients in whom *Candida* colonisation was prospectively assessed, those with a *Candida* score >3 were accurately selected to benefit from early antifungal treatment.^[13]^

The *Candida* colonisation index is the ratio of the number of distinct non-sterile body sites colonised with *Candida* spp. to the number of body sites from which specimens were cultured.^[4]^ An index >0.5 has been shown to have a predictive value of 66% in determining infection. The sensitivity can be further increased if a semi-quantitative fungal load is simultaneously determined. An increasing fungal load in successive specimens is also predictive of invasive infection.^[5]^

Invasive infection by different *Candida* spp. may result in variable outcomes, which makes identification to species level essential.^[12]^ In a small case series of 19 patients admitted with fungal bloodstream infections to the PICU at Inkosi Albert Luthuli Central Hospital, Durban, *C. albicans* was identified as the most common organism, followed by *C. parapsilosis* (Table 1). Of these 19 isolates, 12 were sensitive to fluconazole (63.2%). All patients were on broad-spectrum antibiotics for at least 7 days. Invasive fungal infection was confirmed 48 hours post ICU admission in all but 3 cases (15.8%).

**Table 1:** Prevalence and in-hospital case fatality associated with *Candida* spp. bloodstream infections in the paediatric intensive care unit, Inkosi Albert Luthuli Central Hospital, 2015 and 2016 (*N*=19)*

****	**Prevalence,**	**In-hospital case**
**Organism**	***n* (%)**	**fatality, *n/N* (%)**
*Candida* *albicans*	7 (36.8)	2/7 (28.6)
*Candida* *parapsilosis*	6 (31.6)	1/6 (16.7)
*Candida* *tropicalis*	2 (10.5)	1/2 (50.0)
*Candida* *sake*	1 (5.3)	1/1 (100)
Other *Candida* spp. (unspecified)	3 (15.8)	2/3 (66.7)

*Unpublished data

## Aspergillosis

*Aspergillus* is the most commonly isolated invasive mould, although there are no epidemiological data for this organism in South Africa (SA). Arendrup *et al*.^[9]^ suggested that the incidence of invasive aspergillosis in children was increasing, similar to trends observed in adults; aspergillosis results in a case fatality rate of more than 50%. There are conflicting results in the literature regarding the species distribution of *Aspergillus* among paediatric cases of invasive aspergillosis. In both children and adults, *Aspergillus* *fumigatus* was the most frequently isolated species, followed by *Aspergillus* *flavus*. ^[4,9]^ Most children with invasive aspergillosis present with pulmonary aspergillosis but dissemination to other sites is also seen, particularly to the central nervous system. The aspergillosis clinical syndrome depends on the host’s immune status, ranging from invasive aspergillosis to tracheobronchitis, aspergilloma and chronic necrotising aspergillosis; colonisation without infection also occurs.^[4]^

Several underlying diseases and their treatments are risk factors for invasive *Aspergillus* infection, including haematological malignancies (primary or relapse), allogeneic bone marrow transplantation, granulocytopenia, systemic corticosteroids, immunosuppressive therapies and immunodeficiencies, such as seen in chronic granulomatous disease, severe combined immunodeficiency and organ transplantation (e.g. heart-lung transplantation).^[4]^ The incidence of invasive *Aspergillus* infection varies according to the underlying disease and is highest in immunocompromised children with either acute myeloid leukaemia (5.35%) or acute lymphoblastic leukaemia (1.5%).^[8,16]^ The numbers of patients with chronic obstructive pulmonary disease, influenza or decompensated cirrhosis are increasing and are understudied populations at risk for invasive pulmonary aspergillosis in the ICU.^[17]^ There are limited data for aspergillosis in PICU patients.^[17]^

The clinical presentation of invasive aspergillosis and the rate at which the disease progresses vary.^[16]^ As immunosuppression increases, so does the rate of disease progression.^[16]^ Paediatric patients with invasive *Aspergillus* infection have a 20% higher mortality risk and a 13.5-fold increase in relative risk for death compared with children without invasive *Aspergillus* infection.^[4]^ In one study, the case fatality rate was 53% and multivariable analysis showed that allogeneic haematopoietic stem cell transplantation was a predictor of poor prognosis.^[8]^ The mortality rate from disseminated aspergillosis is very high (up to 80% in those affected). The mortality rate in those with central nervous system involvement, bone marrow transplantation and advanced HIV infection is 88%, 87% and 86%, respectively.^[4]^

## Diagnostic tests for invasive fungal infections

There are limited data on the value of non-culture diagnostic tests in children. Blood cultures are essential diagnostic tests for Candidaemia but are not useful for aspergillosis.^[10]^ The galactomannan test has low specificity and sensitivity and is not recommended for diagnosis of invasive candidiasis.^[10]^ The 1,3-β-D-glucan (BDG) test can be used to exclude invasive fungal infections, including candidiasis, with a sensitivity and specificity of 65% and 80%, respectively.^[10]^ The test is not specific for *Candida* spp. because the antigen is present in many fungal species. The BDG and galactomannan tests are associated with high false-positive rates. The levels of BDG were found to be higher in subjects receiving human blood products, antibiotics and corticosteroid therapy than in those without these treatments.^[18]^ Invasive aspergillosis may be asymptomatic in up to one-third of patients, and diagnostic difficulties are compounded by the lack of characteristic symptoms and accurate diagnostic tests.^[16]^

## Role of prophylaxis

The impact of antifungal therapy on the outcome of invasive fungal infections is affected by the appropriateness and timing of initiation of treatment. Prophylactic antifungal agents are recommended in only a few specific situations and most treatments are administered on an empiric basis.^[19]^ For extremely premature neonates, the use of fluconazole prophylaxis is an attractive option for reducing invasive candidiasis.^[9]^ This is not routinely recommended in the South African setting because of the reported resistance of *Candida* in paediatric units. Govender *et al*. ^[20]^ reported that more than half of the *C. parapsilosis* isolates from bloodstream infections in 2009 - 2010 tested resistant to fluconazole. The guidelines of the European Society for Clinical Microbiology and Infectious Diseases no longer recommend fluconazole for treatment of invasive candidiasis and now endorse the use of echinocandins as first-line empiric therapy.^[19,20]^

Reinforcement of the intestinal mucosal barrier by administration of commensal bacteria (probiotics) as supplements may be useful for prevention of nosocomial fungal infections.^[12]^ Probiotics modify the enteric microflora by colonising the gastrointestinal tract and reduce overgrowth of pathogens that could otherwise lead to colonisation and invasive infection.^[12]^ Some trials have reported a beneficial effect of probiotics in the prevention of enteric colonisation by *Candida* spp. in preterm newborns, but no such trials have been conducted in critically ill paediatric patients.^[12]^

Monitoring for colonisation with *Candida* spp. in children undergoing treatment for severe sepsis or septic shock in the PICU for longer than 5 days may offer an opportunity for early intervention to prevent Candidaemia.^[21]^ Singhi *et al*.^[21]^ demonstrated that 90% of the patients who developed Candidaemia were colonised by the same *Candida* species. Shorter courses of antibiotic therapy and routine surveillance cultures for *Candida* spp. are recommended.

The routine use of antifungal prophylaxis in the general ICU setting is discouraged.^[22]^ The principal negative aspect of prophylaxis is selection of resistant strains and antifungal agent-related toxicities. This problem can be minimised by having better diagnostic tools for invasive fungal infection. The value of prophylaxis against invasive aspergillosis in the intensive care setting remains uncertain.^[16]^

## Future research priorities

More data are required on predisposing factors for fungal infections in critically ill children and the effect of prophylactic antifungal therapy, together with an assessment of the impact on morbidity and mortality. More research is required on prediction rules and diagnostic tests to help with early identification and adequate prophylactic therapy or preemptive therapy. Well-controlled prospective trials are required to assess the changing microbial milieu in PICUs and the impact of antibiotic stewardship on reducing fungal infection in the PICU.

## Conclusion

*Candida* and *Aspergillus* spp. are the most frequently identified fungi in critically ill children, although there are no data on aspergillosis in the SA context. The attributable mortality of these two invasive infections differs mainly because of heterogeneity in the patient populations. The impact of antifungal therapy is affected by the appropriateness and timing of initiation. It is important to identify patients at risk of developing fungal infection early and to draft policies regarding empirical antifungal therapy. There is need for more data to address the role of antifungal chemoprophylaxis in the PICU setting.
